# Evolving Diversity of Hepatitis C Viruses in Yunnan Honghe, China

**DOI:** 10.3390/ijms17030403

**Published:** 2016-03-18

**Authors:** Lanhui Yang, Chenyan Jiang, Song Hu, Qiongni Diao, Jia Li, Wei Si, Mei Chen, Richard Y. Zhao

**Affiliations:** 1 Department of Clinical Laboratory, The First People’s Hospital of Honghe, Mengzi 661100, China; sw85473440@163.com; 2 Division of Molecular Pathology, Department of Pathology, University of Maryland School of Medicine, Baltimore, 21201 MD, USA; 3 Division of Life Science, College of Life Science and Technology, Honghe University, Mengzi 661100, China; jcy_bilogy@126.com (C.J.); 18708733380@163.com (Q.D.); 4 Department of Infectious Diseases, The First People’s Hospital of Honghe, Mengzi 661100, China; Ynhhhs@126.com (S.H.); 13887378618@163.com (J.L.); chenmei120@163.com (M.C.)

**Keywords:** HCV, genotype, subtype, phylogenetic analysis, diversity, Honghe Autonomous Prefecture, Yunnan, China

## Abstract

The Chinese Honghe Autonomous Prefecture (Honghe) in Yunnan Province is a unique ethnic area because it is inhabited by more than ten different minority ethnic groups. Geographically, Honghe directly shares a border with Vietnam. The objective of this study was to investigate genetic diversity and distribution of the Hepatitis C virus (HCV) in Honghe. Ninety nine subjects who were infected with HCV or HCV/HIV (Human Immunodeficiency Virus Type 1) were recruited into this study. HCV genotypes and subtypes were determined based on the sequences of the core/envelope 1 (*C/E1*) and the nonstructural protein 5B (*NS5B*) genomic regions. The viral diversity and origins of dissemination were examined by phylogenetic analyses. Three HCV genotypes (1, 3 and 6) with six subtypes (1b, 3b, 3a, 6a, 6n and 6v) were identified. The most predominant form was genotype 3 (54.6%) followed by 6 (34.3%), and 1 (9.1%). The HCV subtype 3b appeared to be the most frequent form (38.4%) followed by 6n (20.2%) and 3a (16.2%). Statistical analyses suggested a possible rise of the genotype 6a in Honghe among intravenous drug users with HCV/HIV co-infections. Further phylogenetic analyses suggested that similar HCV-6a viruses might have been circulating in the Honghe area for more than a decade, which likely originated from Vietnam or vice versa. Two HCV samples with single HCV infection (SC34 and SC45) were isolated that could represent new recombinant variants. Although the genetic prevalence of HCV in Honghe is in general agreement with that of Southwest China and Yunnan Province, the diversity of HCV genotypes and subtypes in Honghe is somewhat unique and evolving. Information presented here should provide useful information for future health surveillance and prevention of HCV infection in this area.

## 1. Introduction

Hepatitis C virus (HCV) is the major cause of chronic hepatitis, liver cirrhosis and hepatocellular carcinoma (HCC). HCV is a blood-borne virus and the most common routes of HCV transmission are intravenous drug use, sex and mother-to-child infection. There are more than 150 million people globally with chronic HCV infection [1,2]. HCV accounts for approximately 27% of cirrhosis and 25% of HCC worldwide [3]. HCV infection is therefore a major health problem and causes significant morbidity and mortality.

HCV is a positive-sense, single-stranded RNA virus that belongs to the flavivirus category. Its genome is approximately 9600 nucleotide bases (bp), which consists of a 5′ non-coding region (5′ NCR), a single open reading frame (ORF) and a 3′ untranslated region (3′ UTR). The ORF of HCV encodes at least 11 proteins including 3 structural proteins (Core, E1 and E2), a small protein p7, and 6 nonstructural proteins (NS2, NS3, NS4A, NS4B, NS5A and NS5B) [4].

HCV exhibits a high degree of genetic diversity. So far, seven different HCV genotypes with more than 67 of different subtypes have been reported [5]. On average, HCV genotypes differ by 31%–35% of their nucleotide sequences over the entire genome [6], and the subtypes typically differ from each other by at least 15% of nucleotide sequence homology within the coding region [7]. Currently, one of the most common and accepted methods for HCV genotyping is comparing the genetic diversity of two genomic regions, *i.e.*, core/envelope 1 (*C/E1*) and the nonstructural protein 5B (*NS5B*), that are divergent enough to allow the discrimination of HCV genotypes and subtypes [8,9]. The Core gene encodes a core (capsid) protein and the E1 gene encode an envelope protein, which is highly glycosylated and important for cell entry. The NS5B protein is a viral RNA dependent RNA polymerase. It initiates HCV viral replication by using the viral positive RNA strand as its template and it plays a key function in viral replication [10,11,12].

The HCV genome is highly diversified primarily because the HCV genome mutates frequently, which is attributed to its highly error-prone RNA-dependent RNA polymerase. Once an infection has been established, HCV undergoes error-prone replication and likely generates a range of genetic variations, making it difficult to create a preventive vaccine against them [13]. Thus it is imperative to determine the HCV genotypes either for epidemiological surveillance of HCV dissemination or for medical treatment of HCV infections. Besides the high error rate of RNA-dependent RNA polymerase, the pressure exerted by the host immune responses could also drive the evolution of HCV into highly diversified genotypes and subtypes [5]. The genotypes 1 to 3 have a worldwide distribution [2]. Among them, HCV subtypes 1a and 1b are the most common genotypes in the United States, Europe, Japan and Central China. Genotypes 2a and 2b are common in North America, Europe and Japan, representing 10%–30% of the global HCV populations, while genotype 2c is disseminated in Northern Italy [14,15,16,17,18]. The genotype 3 is prevalent in South China and the southern Asian countries. It is variably distributed in other countries [18,19,20]. The HCV genotype 3a is particularly prevalent in intravenous drug users (IDUs) in Europe and the United States [21]. The genotype 4 is distributed in North Africa and the Eastern Mediterranean countries [2,22]. The genotype 5 is highly prevalent in South Africa [23]. The genotype 6 is mainly prevalent in some of the Southeast Asian countries and southwest of China [24,25]. In general, genotype 6 viruses show the highest diversity among HCV. To date, the genotype 6 has 24 different subtypes (6a—6xa) with many unassigned variants. The genotype 7 was only found in an emigrant from Congo [7]. Therefore, distributions of HCV genotypes and subtypes vary significantly by geographic regions.

In China, HCV 1a and 1b are most common in Central China. Genotype 3 is prevalent in Southern China, and genotype 6 is mainly disseminated in the southwest of China such as Yunnan province, where Honghe is.

Different HCV genotypes also vary in their responses to interferon/ribavirin combination therapies. For example, response to interferon treatment dependents on the HCV genotype and subtype. The genotypes of HCV-1 and 4 are typically less responsive to interferon-based treatment than the HCV-2, 3, 5 and 6 [6]. In addition, patients with genotype 1 (particularly the subtype b) and 4 virus may benefit from longer courses of therapy [26]. However, patients with chronic HCV genotype 1b infection exhibit more severe liver disease than patients infected with other genotypes. Therefore, knowing a patient’s HCV viral genotype is critical and clinically important for choosing an appropriate therapy [27,28,29,30]. Information about the distribution of HCV genotypes is also essential for development of HCV vaccines because vaccines raised against antigens from multiple HCV genotypes might be necessary for ultimate global protection of patients with HCV infections. Similarly, development of regional or national-specific strategies to treat patients with HCV infections requires detailed understanding of local dynamics of HCV genotypes and subtypes. Therefore, the objective of this study was to investigate genetic diversity and distribution of HCV in Honghe Autonomous Prefecture (Honghe), Yunnan Province, China.

The area of Honghe was chosen for this study because Honghe is a unique area where, besides the Han majority ethnic group, it is also inhabited by more than ten different minority ethnic groups including Hani and Yi. Moreover, Honghe is in a geographically distinct location because it is on the southern border of Yunnan province, which directly borders Vietnam, where HCV and HIV are in high prevalence.

In this study, HCV genotypes and subtypes were first identified in HCV patients with or without HIV co-infection. Diversity and distribution of the HCV genotypes and subtypes were examined by phylogenetic analysis. HCV genomic similarity and possible origins were studied by phylogenetic analyses based on two of the HCV genomic coding regions (*NS5B* and *C/E1*). Possible significance and new trends of HCV infection in the area of Honghe were discussed.

## 2. Results

### 2.1. Diversity and Prevalence of Hepatitis C virus (HCV) Genotypes and Subtypes in Honghe

The objective of this study was to evaluate the prevalence of HCV genotypes and subtypes in Honghe, Yunnan Province, China. Serum samples were collected from a total of 99 HCV and HCV–HIV co-infected patients who visited the First People’s Hospital of Honghe during the year of 2014. The HCV genotypes and subtypes were first identified using a Sanger sequencing method based on two of the HCV genomic areas of *C/E1* and *NS5B* as previously described [9]. The identified HCV genotypes and subtypes were verified by phylogenetic comparison of the testing sequences with known reference sequences.

Table 1A shows HCV single infection (SC) and Table 1B shows HCV and HIV co-infections (CC). As shown, HCV genotypes and subtypes were successfully determined in most of the samples with the coverage of bi-directional nucleotide sequencing of *C/E1* and *NS5B*, respectively. Others were determined based on either partial coverage of the two gene targets (SC15 and SC23) or one of the gene targets (SC18, SC24, CC5, CC11, CC15, CC22, CC24 and CC26). Interestingly, neither HCV genotype nor subtypes were assigned to the sample of SC34 based on the sequencing results of *C/E1* and *NS5B*, although they were indeed confirmed to be HCV. Similarly, based on the *C/E1* sequence, SC45 was assigned to the subtype 6v, whereas no specific subtype was assigned based on the *NS5B* determination. In addition, two viral variants were detected in SC13 and CC18, suggesting dual infection as previously reported [31,32].

**Figure 1:**
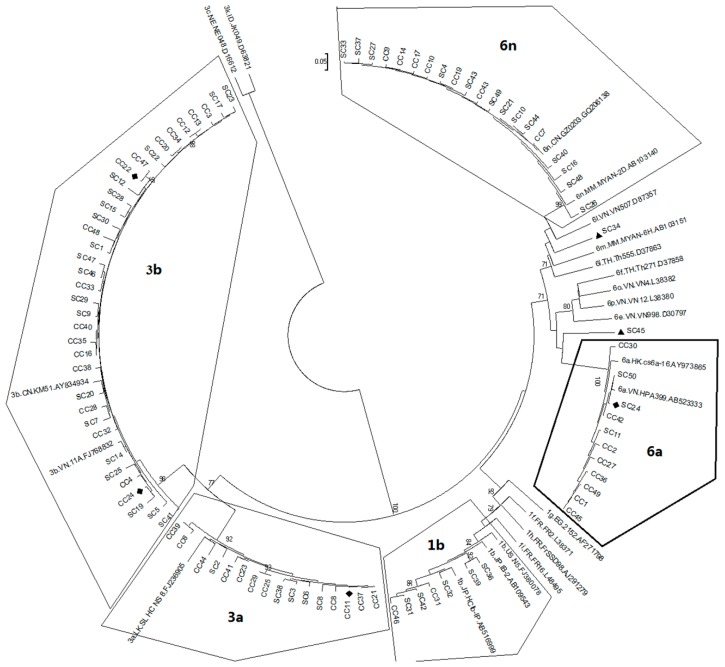
Phylogenetic distribution of Hepatitis C virus (HCV) genotypes and subtypes of the Honghe patients based on comparison of the *NS5B* gene region. The phylogenetic tree was generated based on comparison of the *NS5B* gene region between all of the Honghe sequences and some of the representative HCV genotypic references. The sequence of *NS5B* corresponds to nucleotides of 8276–8615 in the HCV H77 genome (NC_004102). Only three HCV genotypes 1, 3, and 6 were detected as indicated. The number at each node represents the percentage of bootstrap support for 1000 replicates. Only the values of over 70% are shown here. The scale bar represents 0.05 nucleotide or amino acid substitutions per site. SC, single HCV infection; CC, HCV/HIV co-infections. Samples of the SC34 and the SC45 (marked with ▲) formed two diverse clusters with low bootstrap value and low nucleotides similarity, which suggests the subtype assignments of these samples were not clear. All of the assignments of HCV genotypes and subtypes are consistent with the results shown in Figure 2 with the exception of SC34 and SC45. The samples of SC24, CC11, CC22 and CC24 denoted with ♦ were assigned based only on the information of the nonstructural protein 5B (*NS5B*) gene region because sequence information of the core/envelope 1 (*C/E1*) and region was not available due to failures of either PCR or sequencing results.

**Figure 2:**
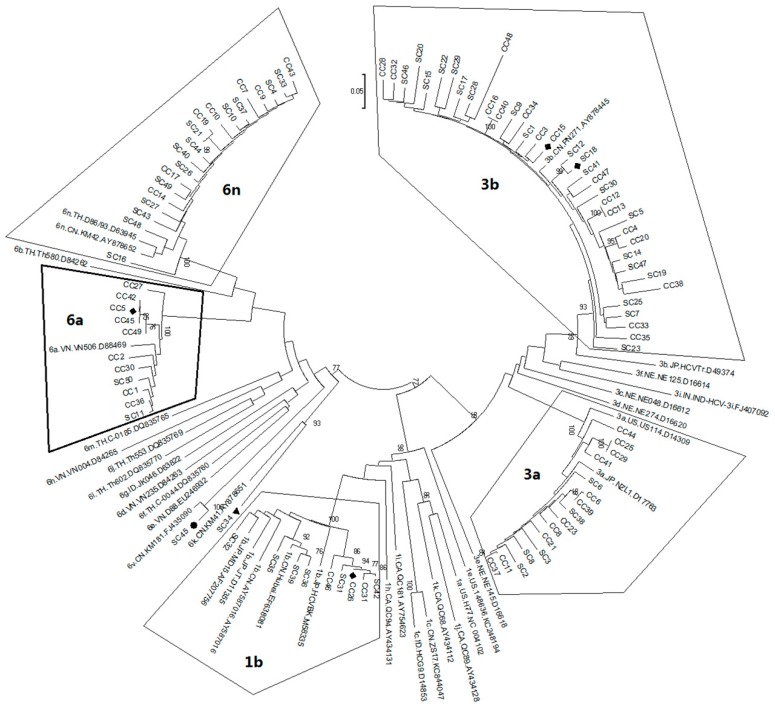
Phylogenetic distribution of HCV genotypes and subtypes of the Honghe patients based on comparison of the *C/E1* gene region. The phylogenetic tree was generated based on comparison of the *C/E1* gene region between all of the Honghe sequences and some of the representative HCV genotypic references. The sequence of *C/E1* corresponds to nucleotides of 869–1, *i.e.*, 292 in the HCV H77 genome (NC_004102). Only three HCV genotypes 1, 3, and 6 were detected as indicated. The number at each node represents the percentage of bootstrap support for 1000 replicates. Only the values of over 70% are shown here. The scale bar represents 0.05 nucleotide or amino acid substitutions per site. Sample of the SC45 (marked with ●) was assigned to subtype 6v with high bootstrap value and high nucleotide similarity. The SC34 (marked with ▲) formed two diverse clusters with low bootstrap values and low nucleotides similarities, which suggests that the subtype assignment of this sample was not clear. All of the assignments of HCV genotypes and subtypes are consistent with the results shown in Figure 1 with the exception of the SC34 and the SC45. The samples of SC18, CC5, CC15 and CC26 denoted with ♦ were assigned based only on the information of the *C/E1* gene region because sequence information of the *NS5B* region was not available due to failures of either PCR or sequencing results.

To confirm the assigned HCV genotypes/subtypes, the MEGA 6 phylogenetic analysis was carried out by comparing each of the testing sequences with known reference sequences that were deposited in the database of the Los Alamos National Laboratory [33]. Overall, assignments of the HCV genotypes and subtypes were consistent with the BLAST search results. Two phylogenetic trees created based on *NS5B* (Figure 1) and *C/E1* (Figure 2) were also in general agreement with each other.

The demographic information and prevalence of the HCV genotypes and subtypes are summarized in Table 2. Specifically, three different HCV genotypes (1, 3 and 6) with six subtypes (1b, 3b, 3a, 6a, 6n and 6v) along with one unassigned genotype 6 were identified. The HCV genotype 3 was the most predominant form with a frequency of 54.6% followed by genotype 6 (34.3%), and genotype 1 (9.1%), respectively. Overall, HCV subtype 3b appeared to be the most frequent form (38.4%) with HCV-6n as the second (20.2%) and HCV-3a the third (16.2%). The gender ratio between male and female patients was about 2:1. However, no statistically significant differences were found between male and female patients with regard to the distribution of HCV genotypes and subtypes. We were unable to evaluate the potential differences of HCV prevalence among different ethnic groups due to the small size of the minority patients. Comparison of the HCV subtype distribution between the patients with single HCV infection and the patients with HCV/HIV co-infections showed no statistical difference (*p* > 0.05). However, statistical difference (*p* = 0.017) was found in the numbers of HCV-6a between the subjects who acquired HCV through blood contact and the IDUs based on a χ^2^ test. Interestingly, all of the 8 IDUs who carried HCV-6a were patients with HCV/HIV co-infections.

Together, genetic analyses of HCV genotypes and subtypes of the patients in Honghe suggested that prevalence of HCV in the Honghe area is in general agreement with that of Yunnan province and southwest China [18,19,20,24,25]. Specifically, the most predominant from of HCV in Honghe was genotype 3, followed by genotypes 6 and 1, respectively, in which HCV subtype 3b was the most frequent form followed by HCV-6n and HCV-3a.

### 2.2. Possible Rise and Dissemination of HCV-6a in Honghe from Vietnam or Adjacent Areas

**Figure 3:**
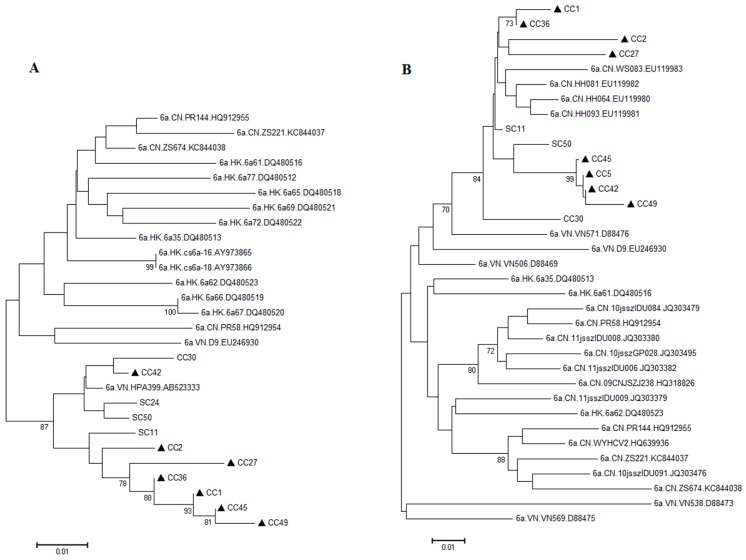
Phylogenetic analysis of HCV genotype 6a. The phylogenetic tree was generated for HCV-6a only. The genetic relationship between the HCV-6a sequences isolated from Honghe and the representative reference sequences of HCV-6a was compared based on the genomic regions of *NS5B* (**A**) and *C/E1* (**B**). All of the references sequences were deposited in the pathogen database of the Los Alamos National Laboratory. The number at each node represents the percentage of bootstrap support for 1000 replicates. The scale bar represents 0.01 nucleotide or amino acid substitutions per site. The bars at the base of the trees indicate the genetic divergence. The two upper case letters added to each of the reference sequences indicate the country origin. CN, China; HK, Hong Kong; VN, Vietnam; SC, single HCV infection; CC, HCV/HIV co-infections; ▲, IDUs.

Although the diversity and prevalence of HCV in Honghe is in general agreement with that of the southwestern part of China, in particular with Yunnan Province, one interesting observation we made was that the HCV-6a subtype appeared to be statistically higher among the IDUs than infections from the other routes (Table 2). Furthermore, all 8 IDUs were from patients with HCV/HIV co-infections, suggesting a possible and unique route of dissemination of HCV-6a. Indeed, when trending analysis was carried out by comparing distribution of HCV genotypes/subtypes reported over time in Yunnan province with that of Honghe, the HCV-6a increased from 5% in 2000–2003, 15% in 2013, and to 20.5% in 2014 (Table 3). These data suggested either the patients in the Honghe area had higher HCV-6a than rest of the Yunnan province or the HCV-6a was on the rise. Thus we were interested in taking a closer look at those IDU cases and probing the possible dissemination sources of HCV-6a. Phylogenetic analysis was carried out to compare all of the HCV-6a sequences isolated from the Honghe patients with all of the HCV-6a reference sequences of *NS5B* or *C/E1*. As shown in Figure 3A, the HCV-6a was typically found in the areas of Hong Kong, Vietnam and China. Interestingly, all of the HCV-6a sequences from Honghe, regardless the routine of transmission, were clustered together and were closely related to a single VN.HPA399.AB523333 sequence, which was originated from an IDU from Hai Phong, Northern Vietnam [34]. Consistently, when the same sequence comparison was analyzed by phylogenetic analysis by using the *C/E1* region, a distribution pattern was also seen (Figure 3B) very similar to that of Figure 3A, *i.e.*, all of the HCV-6a sequences from Honghe were closely clustered together and were linked to a single HCV-6a sequence (VN.VN571.D88476) that came from Vietnam. Also note that there were 4 other HCV-6a sequences that were clustered within the Honghe sequences (CN.WS083.EU119983; CN.HH081.EU119982; CN.HH64.EU119980 and CN.HH093.EU119981). Literature searches on the origins of those sequences revealed that those four HCV-6a sequences were actually isolated either from Honghe (HH) or the nearby Wenshan (WS) area [35].

Therefore, based on these information, it appears that all of the Honghe HCV-6a sequences were derived from a very closely related virus that was circulating in the Honghe area for more than a decade [35], which most likely originated from Vietnam [34].

### 2.3. Possible New HCV Variants of HCV-6 in Honghe

**Figure 4:**
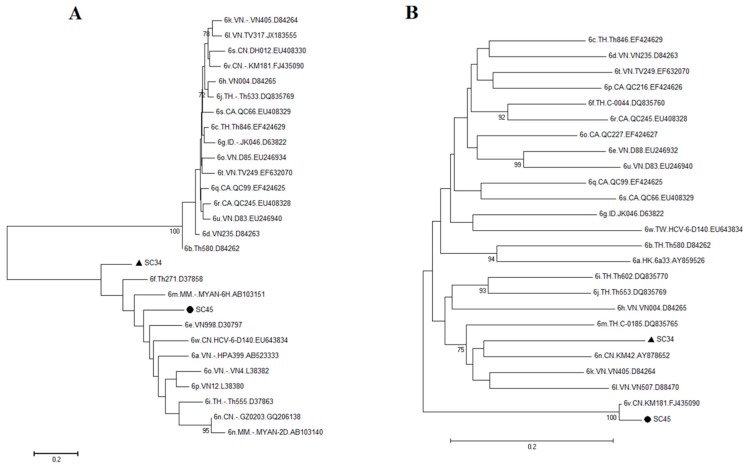
Phylogenetic relationship between SC34 or SC45 with other known subtypes of HCV-6. The genetic relationship between the SC34 or the SC45 isolated from Honghe and the representative reference sequences of HCV-6 subtypes was compared based on the genomic regions of *NS5B* (**A**) and *C/E1* (**B**). All of the references sequences were deposited in the pathogen database of the Los Alamos National Laboratory. The number at each node represents the percentage of bootstrap support for 1000 replicates. The scale bar represents 0.2 nucleotide or amino acid substitutions per site. The bars at the base of the trees indicate the genetic divergence. The two upper case letters added to each of the reference sequences indicate the country origin of that particular sequence. CA, Canada; CN, China; HK, Hong Kong; ID, Indonesia; IN, India; MM, Myanmar; TH, Thailand and VN, Vietnam. SC, single HCV infection. ▲, indicate where SC34 is; ●, indicate where is the SC45.

Besides the HCV-6a genotype described above, two HCV isolates (SC34 and SC45) fell into different HCV-6 genotypes based on the phylogenetic analyses (Figure 4A,B). Alignment of the *C/E1* region of the SC45 sequence showed a close link to HCV-6v.CN.KM 181.FJ435090 with 100% bootstrap value and 95% nucleotide similarity (Figure 4B, marked with ●). The sequence of HCV-6v.CN.KM 181.FJ435090 was from a nearby city of Kunming, Yunnan Province. However, alignment of the *NS5B* region of the SC45 sequence loosely clustered with the Vietnam or Myanmar sequences (Figure 4A, marked with ●), suggesting SC45 could potentially be a new and recombinant variant of HCV-6.

Similarly, alignments of neither *C/E1* nor *NS5B* of SC34 gave rise any clear designation of its subtype, as both sequences diverged by at least 15% of its genetic distance from the nearby sequences (Figure 4A,B). A closer examination and comparison of the SC34 sequences with the reference sequences of the world clearly suggesting that SC34 is genetically distinct from all of those reference sequences based either on the *NS5B* sequences or the *C/E1* sequences (Figure 4A,B). Calculation of the % of sequence homologies of the SC34 with the closest HCV-6 *NS5B* or the *C/E1* reference sequence revealed that it shares 79% or 85% homologies with the *C/E1* or *NS5B* sequences, respectively. By definition, HCV subtypes typically differ from each other by at least 15% in nucleotide sequences within the coding region [7]. Therefore, based on the sequence homology analyses and the phylogenetic analyses of SC34 shown in Figure 4, SC34 was clearly dispersed into two different sub-branches with a low bootstrap value and a significant low nucleotide similarity, suggesting that it might represent a new variant subtype of genotype 6.

## 3. Discussion

In this study, we described the genetic diversity and distribution of HCV in Honghe, Yunnan Province, China. The area of Honghe Autonomous Prefecture is unique because there are more than 10 different ethnic groups living in Honghe, it is in the southeast center of Yunnan province where HCV prevalence is relatively high, and it also borders Vietnam. Three different HCV genotypes (1, 3 and 6) with six subtypes (1b, 3b, 3a, 6a, 6n and 6v) along with one unassigned genotype 6 were identified from this study cohort. The most predominant genotype of HCV in Honghe was 3 with the subtype of 3b, the most frequent form, followed by genotype 6 and 1, respectively. Statistical analyses of the HCV prevalence data the suggested possible rise of genotype 6a in Honghe among the IDUs. Further phylogenetic analyses revealed that all of the Honghe HCV-6a infections might be originated from Vietnam or adjacent areas. Two possible new HCV variants (SC34 and SC45) were isolated that might belong to either a rare 6v (SC45) or a novel recombinant variant (SC34). Additional studies are certainly warranted to confirm the origin of the genotype 6a in Honghe and findings of the possible two new HCV variants.

Although anti-HCV drugs have been discovered that could lead to the cure of HCV infection in some patients [37], HCV infection remains to be a major and growing public health issue in both developing and developed countries [38]. In China, HCV infection was the 4th most commonly reported infectious diseases in 2012 [38]. Four HCV genotypes, 1, 2, 3 and 6, are spreading in China. Among these, the subtypes 1b, 2a, 3a were the most prevalent throughout China [39,40]. Genotypes 3 and 6 showed an increasingly wide geographic distribution [40]. Nevertheless, regional variations of HCV diversity have been extensively reported with active migration of various HCVs across different provinces and regions [41]. For example, HCV 1a and 1b are the most common forms in Central China, whereas genotype 3 is prevalent in South China [18,19,20]. The genotype 6 is disseminated across the entire southwest of China [24,25]. Consistently, the HCV distribution in Yunnan province also follow the pattern of southwest of China with the dominant genotypes of 3, 6 and 1 (Table 3) [25,35]. We showed here that the prevalence of HCV in the Honghe area was in general agreement with that of Yunnan province and southwest of China [18,19,20,24,25]. The HCV subtype 3b was the most frequent form followed by HCV-6n and HCV-3a (Table 1).

Besides the general agreement of HCV diversity with Yunnan province and southwest of China, the diversity of HCV genotypes and subtypes in Honghe was also somewhat unique and evolving. In particular, subtype 6a appeared to be on the rise. For instance, HCV-6a was becoming more prevalent over the years among the IDUs (Table 3). Intriguingly, all of the IDUs carrying HCV-6a were from patients with HCV/HIV co-infections, suggesting a possible common source of transmission. Indeed, four other HCV-6a sequences that were most closely linked to the Honghe patients turned out to be patients detected more than 10 years ago from the same area [35]. Based on further analysis of the phylogenetic trees generated (Figure 3), all of the HCV-6a sequences isolated from Honghe were traced to Vietnam and possibly from a single Vietnam sequence (VN.HPA399.AB523333; Figure 3A), which was originated from an IDU from Hai Phong, Northern Vietnam [34]. Even though we do not know for certain whether those viruses originated from Vietnam or vice versa, one thing that is certain is that there are frequent bilateral trade activities between Honghe and Vietnam. Therefore, these data suggest that there might be close exchanges of HCV-6a viruses between Honghe and Vietnam among those IDUs with HCV/HIV co-infections.

Two possible new HCV viral variants (SC45 and SC34) were detected in the Honghe Autonomous Prefecture. SC45 could potentially be a recombinant variant between the viruses isolated from Kunming (the capital city of Yunnan province) and those detected in Southeastern Asia. This is because the *C/E1* region of SC45 was closely linked to a virus isolated from Kunming (HCV-6v.CN.KM181.FJ435090) and its *NS5B* region was associated with the sequences from Vietnam and Myanmar (Figure 4A, marked with ●). Sequencing of the entire viral genome of SC45 has to take place in order to confirm this possibility.

Based on the information provided by the phylogenetic trees (Figure 4), SC34 might be a new viral variant of genotype 6. This is because SC34 showed very low bootstrap values and very low nucleotide similarities with all of the reference sequences of the *C/E1* and the *NS5B* regions. Therefore, it does not appear to associate closely with any of the reference genotype 6 sequences, which represents its genetic distinction from all of the existing genotype 6 tested. Since the nucleotide substitutions between SC34 and the next closet reference sequences of the *C/E1* and *NS5B* regions were more than 15% (Figure 4), by the definition [7], SC34 might represent a new variant of genotype 6. Our future goal is to confirm this possibility by viral genome sequencing.

In summary, this study describes an evolving pattern of HCV diversity in the Honghe Autonomous Prefecture area, Yunnan Province, China. The HCV study on Honghe is unique because it is a racially high diversified area that is also borders with Vietnam and other southeast countries. The close relationship of the HCV genotypes and subtypes detected in Honghe with its neighboring area such as Vietnam suggested active migration or blood-related exchanges might have taken place among those subjects in these areas. Such a spreading pattern was certainly supported by our observation that the HCV-6a was on the rise particularly among the intravenous drug users with HCV/HIV co-infections. Therefore, the information presented here should provide useful information for future health surveillance and prevention of HCV infection in the Honghe area, Yunnan Province, China.

## 4. Materials and Methods

### 4.1. Study Cohort

A total of ninety nine patients, who visited the Infectious Diseases Clinic of the First People’s Hospital of Honghe from January to November of 2014, were enrolled in this study. All of them met the WS 213-2008 criteria of HCV infection, which was established by the Chinese Ministry of Health. Patients were stratified into 2 groups, *i.e.*, patients with a single HCV infection (SC, Table 1A) or HCV/HIV co-infection (CC, Table 1B). Demographic information of these patients is summarized in Table 2. Sixty seven subjects were male with the age range of 39.0 ± 7.9 years (mean ± SD); 32 were female with the age range of 38.3 ± 8.3 years (mean ± SD). Most of them (82.8%) were in the Han ethnic group, the rest were in the races of Hani, Yi, Hui, Zhuang and others. This study with the project number of KY201409 was conducted with approval of the Medical Ethics Committee of the First People’s Hospital of Honghe. All patients were informed and consented to test HCV viral load. All research samples were normal discarded clinical samples.

### 4.2. Nucleic Acid Extraction and Reverse Transcription

HCV viral RNA was extracted with 200 μL of patient serum by the Tianlong automatic nucleic acids extraction system (Tianlong NP968, Xi’an, China). A 20 μL aliquot was used to measure HCV RNA viral loads by using the Care HCV RT-PCR Assay V2 (Qiagen, Germantown, MD, USA). Samples with viral loads greater than 1 × 10^3^ IU/mL were proceeded to HCV genotyping by RT-PCR for cDNA synthesis. A cDNA reverse transcription reaction consisted of 5 μL of extracted viral RNA, 1 μL of random 6-mers (50 μM), 1 μL of 10 mM dNTPs mix, and 11 μL of nuclease-free water. This mixture was incubated at 70 °C for 5 min, and then placed on ice for at least 2 min. Next, 5 μL of 5 × RT Buffer, 1 μL of M-MLV reverse transcriptase, 1 μL of RNase inhibitor (40 U/μL) were added, mixed gently and incubated at 37 °C for 60 min. The synthesized cDNA was stored at −80 °C prior to PCR.

### 4.3. PCR Amplification

Two HCV genomic regions, Core/E1(*C/E1*) and *NS5B*, were amplified by nested-PCR with 4 pairs of target-specific primers [9] (for details, see Table S1). PCR was conducted with a total volume of 50 μL, which contains 5 μL of 10× Ex Taq buffer (20 mM Mg^2+^ plus), 4 μL of dNTPs mixture (2.5 mM each), 0.25 μL of TaKaRa Ex Taq (5U/μL), 1 μL of 1U TaKaRa Ex Taq HS DNA Polymerase, Outer primer (10 μM), 1 μL of Inner primer (10 μM), and 1 μL of cDNA. The final volume was added up to 50 μL with sterilized distilled water. After activation of the Taq polymerase at 95 °C for 10 min, PCR was carried out in a AB7300 thermal cycler (Applied Biosystems, Foster City, CA, USA) for 40 cycles, each cycle consisting of denaturation at 95 °C for 1 min, annealing at 48 °C (*C/E1*)/50 °C (*NS5B*) for 1 min, and extension at 72 °C for 1 min, followed by a final 5 min extension at 72 °C. A HCV positive control and negative control (water without DNA) were included in each batch of PCR reactions and amplification. The PCR products were then loaded to a 1% agarose gel, which were stained with ethidium bromide and visualized under UV illumination Image system (Kodak Gel logic 440, Rochester, NY, USA). The expected size of the PCR amplified *C/E1* and *NS5B* products were 468 and 388 bp, respectively (a representative image showing results of the agarose gel electrophoresis can be found in Figure S1).

### 4.4. HCV Nucleotide Sequencing and Phylogenetic Analysis

PCR amplified products were subject to Sanger-based nucleotide sequencing that was performed by Shanghai Invitrogen Inc. HCV genotypes and subtypes of the obtained nucleotide sequences were initially generated by using a DNA homology BLAST search program against the Los Alamos HCV database, which is available at http://hcv.lanl.gov/content/sequence/HCV/ToolsOutline.html [42]. Phylogenetic trees were generated by use of a Molecular Evolutionary Genetics Analysis (MEGA) version 6.06 program with the Neighbor–Joining method based on Kimura’s 2-parameter distance Matrix Statistical Analysis [33]. Accession numbers of the reference sequences that were used for the HCV genotype and subtype designations are included as Table S2.

Please note that, the first two letters of a HCV nucleotide sequence were added to name each of the reference gene sequences for easy recognition of the country of origin where the virus was originally discovered [43,44]. For example, VN is generally associated with Vietnam. Others are CA, Canada; CN, China; EG, Egypt; FR, France; HK, Hong Kong; ID, Indonesia; ID, India; JP, Japan; LK, Sri Lanka; MM, Myanmar; NE, Niger; TH, Thailand; TW, Taiwan; and US, USA.

### 4.5. Statistical Analysis

A Pearson *χ*^2^ test or a Fisher’s Exact Test was used using a SPSS software (version 17.0 SPSS, IBM, Endicott, NY, USA) to determine possible significances between the HCV genotype and subtype frequency among the different groups. Probability (*p*) values of 0.05 or less were considered statistically significant.

## Figures and Tables

**Table 1:** (**A**) Determination of Hepatitis C virus (HCV) genotypes and subtypes based on sequencing results of core/envelope 1 (*C/E1*) and the nonstructural protein 5B (*NS5B*) in the single HCV infection group; and (**B**) Determination of HCV genotypes and subtypes based on sequencing results of *NS5B* and *C/E1* in the HCV and HIV-1 co-infection group.

(A) Single HCV Infection Group								
Sample	*NS5B*	*C/E1*	Sample	*NS5B*	*C/E1*	Sample	*NS5B*	*C/E1*
SC1	3b	3b	SC18	nd	3b	SC35	1b	1b
SC2	3a	3a	SC19	3b	3b	SC36	1b	1b
SC3	3a	3a	SC20	3b	3b	SC37	6n	6n
SC4	6n	6n	SC21	6n	6n	SC38	3a	3a
SC5	3b	3b	SC22	3b	3b	SC39	1b	1b
SC6	3a	3a	SC23	3b	3b	SC40	6n	6n
SC7	3b	3b	SC24	6a	nd	SC41	3b	3b
SC8	3a	3a	SC25	3b	3b	SC42	1b	1b
SC9	3b	3b	SC26	6n	6n	SC43	6n	6n
SC10	6n	6n	SC27	6n	6n	SC44	6n	6n
SC11	6a	6a	SC28	3b	3b	SC45	U	6v
SC12	3b	3b	SC29	3b	3b	SC46	3b	3b
* SC13	3b	6a	SC30	3b	3b	SC47	3b	3b
SC14	3b	3b	SC31	1b	1b	SC48	6n	6n
SC15	3b	3b	SC32	1b	1b	SC49	6n	6n
SC16	6n	6n	SC33	6n	6n	SC50	6a	6a
SC17	3b	3b	SC34	U	U	–	–	–
Total	50							
**(B) HCV and HIV Co-Infection Group**								
**Sample**	***NS5B***	***C/E1***	**Sample**	***NS5B***	***C/E1***	**Sample**	***NS5B***	***C/E1***
CC1	6a	6a	* CC18	3b	6a	CC35	3b	3b
CC2	6a	6a	CC19	6n	6n	CC36	6a	6a
CC3	3b	3b	CC20	3b	3b	CC37	3a	3a
CC4	3b	3b	CC21	3a	3a	CC38	3b	3b
CC5	nd	6a	CC22	3b	nd	CC39	3a	3a
CC6	3a	3a	CC23	3a	3a	CC40	3b	3b
CC7	6n	6n	CC24	3b	nd	CC41	3a	3a
CC8	3a	3a	CC25	3a	3a	CC42	6a	6a
CC9	6n	6n	CC26	nd	1b	CC43	3a	3a
CC10	6n	6n	CC27	6a	6a	CC44	6n	6n
CC11	3a	nd	CC28	3b	3b	CC45	6a	6a
CC12	3b	3b	CC29	3a	3a	CC46	1b	1b
CC13	3b	3b	CC30	6a	6a	CC47	3b	3b
CC14	6n	6n	CC31	1b	1b	CC48	3b	3b
CC15	nd	3b	CC32	3b	3b	CC49	6a	6a
CC16	3b	3b	CC33	3b	3b	–	–	–
CC17	6n	6n	CC34	3b	3b	–	–	–
Total	49							

Note: nd: not determined due to PCR-amplification failure; –: blank space; U: Unassigned subtype; * two viral variants were detected, suggesting dual infection as previously reported [31,32], and they are verified by fluorescence PCR with HCV subtype-specific probes (Triplex International Biosciences, China). SC: single HCV infection; CC, HCV/HIV co-infection.

**Table 2:** Demographic information and HCV genotypes and subtypes distribution of the study cohort.

Characteristics/Subtypes	Sample Numbers (%)								Total
	1b	3a	3b	6n	6a	6v	Unassigned	Dual-Infection	
**Gender**									
Male	6 (9.0)	13 (19.4)	27 (40.3)	10 (14.9)	9 (13.4)	1 (1.5)	1 (1.5)	–	67
Female	3 (9.4)	3 (9.4)	11 (34.4)	10 (31.2)	3 (9.4)	–	–	2 (6.2)	32
Total	9 (9.1)	16 (16.2)	38 (38.4)	20 (20.2)	12 (12.1)	1 (1.0)	1 (1.0)	2 (2.0)	99
**Races**									
Han	7 (8.5)	15 (18.3)	32 (39.0)	16 (19.5)	10 (12.2)	1 (1.2)	–	1 (1.2)	82
Hani	1 (10.0)	1 (10.0)	3 (30.0)	4 (40.0)	–	–	–	1 (10.0)	10
Yi	–	–	2 (50.0)	–	2 (50.0)	–	–	–	4
Others	1 (33.3)	–	1 (33.3)	–	–	–	1 (33.3)	–	3
**Route of Transmission**									
Blood Contact	5 (10.4)	5 (10.4)	20 (41.7)	13 (27.1)	2 (4.2) *	1 (2.1)	1 (2.1)	1 (2.1)	48
IDU	2 (5.1)	7 (17.9)	15 (38.5)	6 (15.4)	8 (20.5) *	–	–	1 (2.6)	39
Sex	2 (18.2)	4 (36.4)	3 (27.3)	1 (9.1)	1 (9.1)	–	–	–	11
Vertical	–	–	–	–	1 (100)	–	–	–	1
**HIV Infection**									
Yes	3 (6.1)	11 (22.4)	18 (36.7)	7 (14.3)	9 (18.4)	–	–	1 (2.0)	49
No	6 (12.0)	5 (10.0)	20 (40.0)	13 (26.0)	3 (6.0)	1 (2.0)	1 (2.0)	1 (2.0)	50

Note *: χ^2^ value 5.625, *p* = 0.017, *i.e.*, *p* < 0.05. Statistical significant difference was detected with the distribution of HCV subtype 6a between blood contact and intravenous drug users (IDUs). Others: other ethnic groups such as Zhuang and Hui races.

**Table 3:** Comparison of HCV genotypes and subtypes distribution over time within Yunnan province and with Vietnam among IDUs.

HCV Subtypes	Yunnan (%) *n* = 80	Yunnan (%) *n* = 100	Honghe (%) *n* = 39	Vietnam (%) *n* = 145
Genotype 1	21.2	12.0	5.1	70.0
1a	1.2	2.0	0	42.0
1b	20.0	10.0	5.1	28.0
Genotype 3	53.8	41.0	56.4	2.0
3a	23.8	12.0	17.9	1.0
3b	30.0	29.0	38.5	1.0
Genotype 6	25.0	47.0	35.9	28.0
6a	5.0	15.0	20.5	22.8
6n	11.2	30.0	15.4	0
6 others	8.8	2.0	0	5.5
Year of Study	2000–2003	2013	2014	2008–2009
References	[35]	[25]	This study	[36]

## Supplementary Materials

Supplementary materials can be found at http://www.mdpi.com/1422-0067/17/3/403/s1.
