# Supplementary material for: Evolving Diversity of Hepatitis C Viruses in Yunnan Honghe, China
Source: Int J Mol Sci. 2016 Mar 18;17(3):403. doi: 10.3390/ijms17030403 (PMC4813258; doi:10.3390/ijms17030403)
Supplement: Supplementary file 1 [file ijms-17-00403-s001.pdf]

Supplementary Materials: Evolving Diversity of Hepatitis C Viruses in Yunnan Honghe of China

Lanhui Yang, Chengyan Jiang, Song Hu, Qiongni Diao, Jia Li, Wei Si, Mei Chen and Richard Y. Zhao

Table S1. Nucleotide primer pairs of the core/envelope 1 (*C/E1*) and the nonstructural protein 5B (*NS5B*) used in this study. HCV: Hepatitis C virus.

HCV Genomic Region	Polarity and Sequences	Position	Size of PCR Product (bp)
C/E 1 Outer	Sense: 5′-GCAACAGGGAAYYTDCCYGGTTGCTC-3′	834–859	493
	Anti-sense: 5′-GTRGGNGACCARTTCATCATCA-3′	1306–1327	
C/E 1 Inner	Sense: 5′-TDCCCGGTTGCTCTTTYTCTAT-3′	847–868	468
	Anti-sense: 5′-TTCATCATCATGTCCCAGGCCAT-3′	1293–1315	
NS5B Outer	Sense: 5′-CCHATGGGGTTYTCCTAI′GACACCAG-3′	8241–8266	406
	Anti-sense: 5′-GGNGCYGAGTAYCTGGTCATGGC-3′	8625–8647	
NS5B Inner	Sense: 5′-TTCTCRTATGAYACCCGCTGYTTTGA-3′	8250–8275	388
	Anti-sense: 5′-TACCTVGTCATAGCCTCCGTGAA-3′	8616–8638	

Table S2. List of accession numbers of the reference sequences used for HCV genotype and subtype designations.

Accession Numbers of Reference Sequences of HCV Genotype and Subtype
CA.QC66.EU408329; CA.QC68.AY434112; CA.QC89.AY434128; CA.QC94.AY434131; CA.QC99.EF424625; CA.QC181.AY754623; CA.QC245.EU408328; CA.QC216.EF424626; CA.QC227.EF424627; CN.09CNJSZJ238.HQ318826; CN.10jsszIDU084.JQ303479; CN.10jsszIDU091.JQ303476; CN.10jsszGP28.JQ303495; CN.11jsszIDU006.JQ303382; CN.11jsszIDU008.JQ303380; CN.11jsszIDU009.JQ303379; CN.AY587016.AY587016; CN.GZ0203.GQ206138; CN.HH064.EU119980; CN.HH081.EU119982; CN.HH093.EU119981; CN.Hubei.EF638081; CN.KM41.AY878651; CN.KM42.AY878652; CN.KM51.AY834934; CN.KM181.FJ435090; CN.PN271.AY878445; CN.PR58.HQ912954; CN.PR144.HQ912955; CN.WS083.EU119983; CN.WYHCV2.HQ639936; CN.ZS17.KC844047; CN.ZS221.KC844037; CN.ZS674.KC844038; EG.2152.AF271798; FR.FR2.L38371; FR.FR16.L48495; FR.FrSSD98.AJ291279; HK.cs6a-16.AY973865; HK.cs6a-18.AY973866; HK.6a33.AY859526; HK.6a35.DQ480513; HK.6a61.DQ480516; HK.6a62.DQ480623; HK.6a65.DQ480518; HK.6a66.DQ480519; HK.6a67.DQ480520; HK.6a69.DQ480521; HK.6a72.DQ480522; HK.6a77.DQ480521; ID.JK046.D63822; ID.JK049.D63821; IN.HCG9.D14853; IN.IND-HCV-3i.FJ407092; JP.HCIb-IP.AB516999; JP.HCV-BK.M58335; JP.HCV-Tr..D49374; JP.IB-2.AB109543; JP.JT.D11355; JP.MD15.AF207756; JP.NZL1.D17763; LK.SL_HC_NS_8.FJ236905; MM.MYAN-2D.AB103140; MM.MYAN-6H.AB103151; NE.NE048.D16612; NE.NE125.D16614; NE.NE145.D16618; NE.NE274.D16620; TH.C-0044.DQ835760; TH.C-0185.DQ835765; TH.D86/93.D63945; TH.Th271.D37858; TH.Th553.DQ835769; TH.Th555.D37863; TH.Th580.D84262; TH.Th602.DQ835770; TH.Th846.EF424629; TW.HCV-6-D140.EU643834; US.H77.NC_004102; US.N5.FJ380078; US.US114.D14309; VN.D83.EU246940; VN.D88.EU246932; VN.D9.EU246930; VN.HPA399.AB523333; VN.TV249.EF632070; VN.TV317.JX183555; VN.VN004.D84265; VN.VN4.L38382; VN.VN12.L38380; VN.VN235.D84263; VN.VN405.D84264; VN.D9.EU246930; VN.VN506.D88469; VN.VN507.D87357; VN.VN538.D88473; VN.VN569.D88475; VN.VN571.D88476; VN.VN998.D30797.

CA, Canada; CN, China; HK, Hong Kong; ID, Indonesia; IN, India; MM, Myanmar; TH, Thailand and VN, Vietnam. SC, single HCV infection.

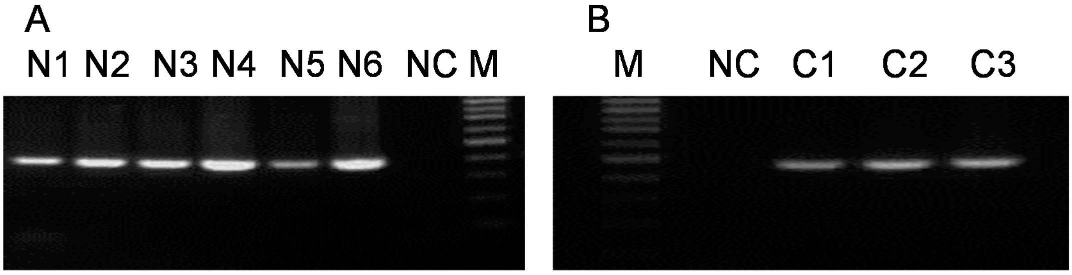

Figure S1. Results of the agarose gel electrophoresis showing PCR amplified gene products of HCV *NS5B* (**A**) and *C/E* (**B**) genes. N1-6 represent some of the PCR amplified gene products of *NS5B*. The expected size is 388 bp. C1-3 show some of the PCR amplified gene products of *C/E*. The expected size is 468 bp. NC: negative control. M: 100bp marker.
